# Wear Performance of a Physical Vapour Deposition-Coated, Spark Plasma Sintered TiB_2_/Ti Composite Lubricated with Externally Introduced hBN at Temperatures up to 900 °C

**DOI:** 10.3390/ma18235274

**Published:** 2025-11-21

**Authors:** Remigiusz Michalczewski, Maciej Łuszcz, Marek Kalbarczyk, Zbigniew Słomka, Edyta Osuch-Słomka, Jarosław Molenda, Le Liu, Maksim Antonov, Irina Hussainova, Manel Rodríguez Ripoll

**Affiliations:** 1 Łukasiewicz Research Network—Institute for Sustainable Technologies, ul. K. Pułaskiego 6/10, 26-600 Radom, Poland; maciej.luszcz@itee.lukasiewicz.gov.pl (M.Ł.); marek.kalbarczyk@itee.lukasiewicz.gov.pl (M.K.); zbigniew.slomka@itee.lukasiewicz.gov.pl (Z.S.); edyta.slomka@itee.lukasiewicz.gov.pl (E.O.-S.); jaroslaw.molenda@itee.lukasiewicz.gov.pl (J.M.); 2 Department of Mechanical and Industrial Engineering, Tallinn University of Technology, Ehitajate 5, 19086 Tallinn, Estonia; le.liu@taltech.ee (L.L.); maksim.antonov@taltech.ee (M.A.); irina.hussainova@taltech.ee (I.H.); 3 AC2T Research GmbH, Viktor-Kaplan-Straße 2/C, 2700 Wiener Neustadt, Austria; manel.rodriguez.ripoll@ac2t.at

**Keywords:** physical vapour deposition coatings, TiB_2_/Ti composites, hexagonal boron nitride, spark plasma sintering, wear analysis, high temperature

## Abstract

In this paper, the achieved state-of-the-art understanding regarding the wear behaviour of various PVD (physical vapour deposition) coatings deposited on TiB_2_/Ti composites produced by SPS (spark plasma sintering) is presented. The objective of this paper is to investigate the wear behaviour of various PVD coatings deposited on TiB_2_/Ti composites manufactured by SPS, when lubricated with hexagonal boron nitride (hBN) as an external solid lubricant in the range from room temperature up to 900 °C in friction contacts under extreme pressure and with oscillation relative motion. Four multicomponent and multilayer coatings were investigated based on AlCrN and TiCrN coatings with TiCrN-AlCrN/AlCrTiN/Si_3_N_4_ interlayers and various external layers (AlCrN, Si_3_N_4_, AlCrTiSiN, and AlCrTiSiN gradient with increasing oxygen gradient replacing nitrogen). The wear tests were performed by means of a ball-on-disc SRV friction and wear tester using reciprocating motion of the Si_3_N_4_ ball sliding against a coated disc from room temperature up to 900 °C. The best protection against wear and oxidation at higher temperatures (even up to 900 °C) was achieved for coatings with AlCrN and AlTiCrN external layers, and hBN lubricant was used simultaneously.

## 1. Introduction

Recent years have witnessed a surge in interest in new materials specifically designed for applications operating at temperatures up to 1000 °C, e.g., material processing operations (cutting, hot forming, stamping), bearings, automotive parts. In practical applications, the concurrent presence of high contact pressure, oscillation relative motion, and extreme temperatures can result in elevated and unstable frictional forces, culminating in severe wear of interacting surfaces.

In the literature, the possibilities of improving resistance, i.e., new type materials, deposition of modern anti-wear or hard coatings, application of known solid state lubricants, and development of new lubricants, were analysed from various perspectives [1,2,3]. High temperature materials are expected not only to have excellent friction and wear properties, but also to exhibit chemical, corrosion, thermal, and mechanical stability. Given that, comprehensive self-lubricating systems, leveraging the benefits of solid-lubricating compounds to minimise friction and wear at various temperatures, seem to be a promising solution.

The authors posited that titanium alloys and titanium composites were prime candidates for high-temperature applications. They are widely used in the manufacturing of machine parts, owing to their low density and excellent mechanical properties, including corrosion resistance, high strength, and high modulus and fracture toughness [4,5,6]. However, under various conditions, such as dry air or high temperatures, titanium alloys exhibit poor tribological properties [1,7,8], which limits their application. Tribo-oxide layers were formed at high temperatures during our tests. Various results were obtained, depending on the chemical composition of the alloy and on the test conditions.

At high temperatures, tribo-oxide layers formed during tests, but research groups obtained different results, dependent on the chemical composition of an alloy and the test conditions. Under sliding conditions, Ti–6Al–4V and Ti–6.5Al–3.5Mo–1.5Zr–0.3Si alloys exhibit a protective behaviour of the developed tribo-layer [9,10,11,12]. However, brittle vanadium and titanium oxides form out of Ti and Ti–6Al–4V, increasing spallation, which leads to poor tribological behaviour [8,10,11].

Many studies and tests, e.g., laser processing, ceramic reinforcement incorporation [13,14,15,16,17,18], were conducted to determine how the properties of titanium and its alloys can be improved. Mechanical properties were improved through reinforcement with ceramic particles [19]. The additions of titanium boride, carbide, and nitride; boron and silicon carbides; and alumina were studied [14,15,18]. Given its density, chemical and thermodynamical stability similar to titanium, titanium monoboride is one of the most prospective reinforcements [1,18,20].

TiB_w_/Ti composites are manufactured by means of spark plasma sintering (SPS) [1,2,21,22,23], the field-assisted sintering technique (FAST) [24], hot isostatic pressing [25], vacuum sintering [26], laser sintering methods [14,27,28], microwave sintering [29], and, as coatings, e.g., by TIG cladding [30]. Ti and TiB obtained with in situ methods generate excellent interface bonding [1,31]. During sintering, a part of pure titanium or titanium from alloy reacts with boron derived from pure powder or titanium diboride and creates titanium monoboride—a titanium or titanium alloy composite. One such method, which is highly advantageous, is SPS [23,32,33]. Given that a protective atmosphere is used, sintering materials, which are susceptible to oxidation, are likely to sinter. A high densification of material is obtained by applying uniaxial pressure. The use of current flow reduces the required sintering temperature and the process run time. The method enables powders to be sintered at lower temperatures than in pressureless sintering and contributes to obtaining dense, fine-grained materials.

TiB_w_/Ti composites have mainly been studied at room temperature, and limited data are available on friction and wear of this material at an elevated temperature [2,7,13].

The results presented in [2] proved that composites with the TiB_w_:Ti ratio of 50:50 wt% are excellent candidates for high-temperature applications, where good wear resistance is required, especially under sliding conditions. However, this material shows poor wear resistance under oscillation conditions at temperatures exceeding 400 °C [34]. This problem can be solved, for example, by laser incorporation of a nickel-bismuth (Ni-Bi) mixture into Ti-TiB_x_, which can protect the composite from wear in real-life environments, where different types of motion take place, and extend the lubrication temperature range (below 700 °C) of Ti-TiB_2_ composites [35].

Based on the literature review, a titanium composite with titanium boride reinforcement (TiB_2_/Ti) was selected as the substrate material [34,35].

To overcome the limitations of TiB_2_/Ti composites, namely poor wear resistance under oscillating conditions at temperatures exceeding 400 °C [34], the application of physical vapour deposition (PVD) coatings resistant to high temperatures was proposed.

The existing PVD coatings are designed for use mainly on steel surfaces to prevent extreme wear under a wide range of ambient temperatures, and without being optimised for metal-ceramic composite substrates [34].

There is a well-known practice of using protective coatings to improve the wear resistance of materials, e.g., polymer coatings, low-friction carbon-based coatings, or hard (e.g., nitride-based) coatings [36,37,38,39]. Due to the high testing temperature, all organic or carbon coatings should be rejected because of their low oxidation temperature. A modern technology for depositing multifunctional coatings is (PVD) [40,41,42]. An important characteristic of PVD coatings is the fact that their thickness ranges between 1 and 5 µm, i.e., within the dimensional tolerances of typical machine elements [42,43,44].

Therefore, nitride coatings used as covers for cutting tools operating at temperatures exceeding even 1000 °C [27,28,29,30,31] can be deposited on parts ready for use. Among the elements utilised for these coatings are Al, Cr, Si, Ti, V, and W [36,43,44,45,46,47].

A wide range of single-layered coatings (e.g., TiN, CrN), multilayer coatings with combined properties of several materials (e.g., TiC/Al_2_O_3_/TiN), or multi-element coatings such as the TiAlN, BCN, AlCrN, and TiAlYN systems [46,48,49] are available. The modification of coating composition expands their area of application. The presence of Al in classic TiN and CrN coatings increases wear resistance and thermal stability at temperatures up to 1000 °C [48,50,51]. In Ti_x_Al_y_N coatings, a self-adaptation mechanism contributing to maintaining a high wear resistance at elevated temperatures (up to 1000 °C), was observed [49]. The remarkable thermal stability of these coatings has been attributed to the formation of a highly protective two-layer oxide film including a very dense Al_2_O_3_ top layer. Despite the extraordinary wear resistance of the Ti_x_Al_y_N coatings, their frictional performance at high temperatures is still not sufficient. One of the options to ensure lubrication behaviour is through the introduction of components decreasing friction, such as TiAlCrN/TiAlYN or TiAlN+WC/C (a-C:H:W) [52,53]. However, modifications of this kind reduce the temperature resistance by a few hundred Celsius degrees, i.e., the final temperature is below 700 °C.

Only coatings with an appropriate operating temperature range and coatings with high adhesion to the composite substrate can be selected. Such coatings are designed to be deposited on steel, but the analysed composite has different properties than iron alloys, i.e., heterogeneous structure, thermal expansion coefficients of different components, porosity presence, and difficulty preparing the surface. Coatings to be applied on a steel substrate do not always show similar adhesion to the TiB_2_/Ti composite substrate [54]. As a result, adhesion tests must precede the selection of the coating.

Despite recent efforts to understand the lubrication of PVD-coated surfaces, the tribo-chemical action mechanism has not been fully explained so far. In some works, solid lubricants of different types are encapsulated in nano-sized reservoirs inside a coating [55,56].

In this analysis, the PVD coatings were applied on ceramic TiB_2_/Ti composites. A commercially available AlCrN-based coating was chosen as a reference coating based on previous studies [54,57,58]. The coating has a good adhesion to TiB_2_/Ti composites sintered by SPS [54] and improves their wear resistance at high temperatures under dry conditions [34]. Furthermore, it has been proven that the combination of the AlCrN coating and hBN reduces the TiB_2_/Ti composite wear and improves resistance to oxidation at a wide range of operating temperatures (from 25 to 900 °C) [59]. The observed benefits of the application of the commercially available coatings on TiB_2_/Ti composites inspired the use of multi-layer coatings, which offer much greater possibilities than single-layer coatings [60].

Three state-of-the-art multilayer coatings were developed: TiN | TiCrN-AlCrN | AlTiCrN; TiN | TiCrN-AlCrN | AlCrTiN/Si_3_N_4_; and TiN | TiCrN-AlCrN | AlCrTiN/Si_3_N_4_ | AlCrTiSiN with a gradient increase in oxygen content, with AlCrTiSiN instead of nitrogen in the outer layer. Each of the developed coatings consists of an interlayer responsible for interaction with the substrate (TiN), an internal layer (TiCrN-AlCrN, TiCrN-AlCrN plus AlCrTiN-Si_3_N_4_), and an external layer (structure AlCrTiN/Si_3_N_4_, gradient structure AlCrTiSiN+ON, single structure AlTiCrN). Previous research under dry conditions proved that the high temperature wear resistance of the developed multilayer coatings was improved compared with the resistance of the AlCrN-based coating [61]. Further studies are required to investigate the activity of solid lubricants and multilayer coatings at high temperatures.

Solid state lubricants are used in all applications where liquid lubricants cannot be applied [62]. In principle, solid lubricants are designed to function with ferrous surfaces (e.g., steel on steel). However, it is uncertain whether these solid lubricants can effectively reduce friction and wear on non-ferrous surfaces such as TiB_2_/Ti composites and PVD coatings.

Among the numerous available solid lubricants, hexagonal boron nitride (hBN) is favoured to be used in extreme high-temperatures. The hBN phase has a structure analogous to graphite, with hexagonal planes, which are easily sheared layers held by weak van der Waals forces. They ensure low movement resistance and reduce friction [63,64,65,66]. hBN has a high thermal (above 1000 °C) and chemical stability, rendering it one of the most promising solid lubricants for current and future applications [63]. hBN is used as a release agent [64] and has been tested as a solid lubricant [63,64,65,67]. It is applied at various stages of manufacturing.

As a component of a composite, hBN is added at the stage of powder preparation, e.g., of Ni-W+hBN composite powders made by self-heating synthesis (SHS) [67], Al_2_O_3_-cBN-hBN composite powders made by conventional mixing [68] or made in situ by spark plasma sintering from CO(NH_2_)_2_ and H_3_BO_3_ [69]. It is also being investigated as an additional raw powder material for sintering with ceramic matrix [70]. Such composites are sintered using special methods with a protective atmosphere or vacuum [67,68,71], e.g., by means of spark plasma sintering (SPS) or hot pressing (HP). The incorporation of hBN in Ni and Ni-W systems improves hardness, wear, and corrosion resistance [67,72] and it improves hardness and wear behaviour [68] in Al_2_O_3_-cBN systems. However, changes in the base material are not always efficient and may adversely affect mechanical properties [68].

As an additive to coatings, hBN does not affect the base material. Ni-B+hBN composite coatings are deposited on a steel substrate by means of a conventional electroplating method [66,73]. Such coatings have similar protective properties, i.e., wear and corrosion resistance, to solid composites. hBN nanosheets were studied in addition to an epoxy resin-TiO_2_ composite deposited as a layer on 304L stainless steel, which resulted in wear reduction [74]. However, the temperature range above which the properties of TiB_2_/Ti composites need to be improved is higher than the service temperature of these types of coatings [73,74].

The external application of the hBN lubricant to the TiB_2_/Ti composite can be carried out by aerosol spraying or deposition as a suspension-grease (dispersed in solvent) [59,60,70]. The test results confirmed that the use of hexagonal boron nitride as a solid lubricant does not increase the wear resistance of the TiB_2_/Ti composite [59] under high-temperature conditions. For this reason, the application of this lubricant to the tribo-couple is not always efficient. Further research is necessary to study the phenomena occurring in the tribocouple lubricated with hexagonal boron nitride and to explain the correlation between the type of surface, its parameters, and the base material wear characteristics.

The objective of this paper is to investigate the wear behaviour of various PVD coatings deposited on TiB_2_/Ti composites manufactured by SPS, when lubricated with hexagonal boron nitride (hBN) as an external solid lubricant in the range from room temperature up to 900 °C in friction contacts under extreme pressure and with oscillation relative motion.

For this purpose, four types of coating external layers were considered, i.e., a single structure: AlCrN, AlTiCrN; a structure: AlCrTiN/Si_3_N_4_; and a gradient structure: AlCrTiSiN+ON, along with hBN as the solid lubricant. This issue is of great significance for engineers developing new materials with excellent wear resistance at high working temperatures, required in the modern manufacturing industry.

## 2. Materials and Methods

### 2.1. The TiB_2_/Ti Composite

The TiB_2_/Ti composite samples were manufactured using a spark plasma sintering device (SPS) (FCT Systeme GmbH, Sonneberg, Germany) from TiB_2_ (>99.5% Alfa Aesar, Karlsruhe, Germany, ≤44 µm) and pure Ti (Alfa Aesar, Germany, particle size ≤50 µm) powders with the weight ratio of approximately 50–50%. The names of the composites were derived from the base powders. The preparation procedure was based on the previous research findings. Those studies demonstrated that optimal adhesion for coatings was achieved when they were deposited on composites fabricated from a mixture of two powders: pure titanium (Ti) and titanium diboride (TiB_2_) [54].

Substrate powders were conventionally pre-mixed using a mechanical rotating mixer for 2 h. The mixture was loaded into a graphite mould and heated up to the temperature of processing. The process parameters are summarised in Table 1.

Some cylindrical samples with a thickness of 9 mm and a diameter of 25 mm were fabricated using the SPS process. Subsequent to manufacturing, graphite scale was removed. The composite exhibited a hardness of 76 ± 2 HRC. Surface polishing was conducted using an ATM Saphir 550 automatic polisher [54]. It achieved a surface roughness (R_a_) of 0.091 ± 0.008 µm. The surface roughness was further measured with the use of a non-contact 3D profiler. The composite exhibited a porosity of less than 2%. The disparity in mechanical properties between the constituent phases, coupled with the presence of pores, hindered the attainment of a perfectly smooth surface. Furthermore, the polishing process resulted in the exposure of additional pores. The maximum pore depth observed in the composite samples was approximately 2.5 µm.

The phase composition of the manufactured composites was measured with a D8 Discover diffractometer (Bruker AXS GmbH, Karlsruhe, Germany), equipped with a Co X-ray tube (Kα1 = 1.79026 [M2] Å).

### 2.2. Coating Deposition

**Figure 1:**
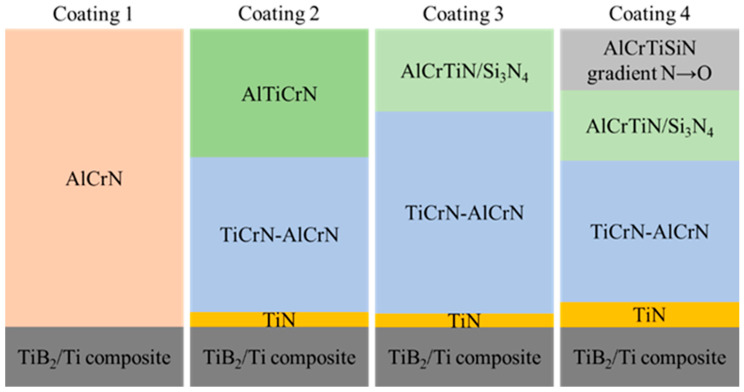
The scheme of coating structures deposited on steel reference samples. Coating 1: AlCrN, thickness: 1.2 µm. Coating 2: TiN, thickness: 0.2 µm; TiCrN-AlCrN, thickness: 2.2 µm; AlTiCrN, thickness: 1.8 µm. Coating 3: TiN, thickness: 0.2 µm; TiCrN-AlCrN, thickness: 3.0 µm; AlCrTiN/Si_3_N_4_, thickness: 1.2 µm. Coating 4: TiN, thickness: 0.5 µm; TiCrN-AlCrN, thickness: 2.8 µm; AlCrTiN/Si_3_N_4_, thickness: 1.4 µm; AlCrTiSiN gradient N→O, thickness: 1.2 µm.

Four types of the coatings were used. The AlCrN coating (Coating 1, thickness: 1.2 µm) is a commercial PVD-method product (BALINIT^®^ ALCRONA PRO made by Oerlikon Balzers, Polkowice, Poland). Coatings 2–4 were deposited on composite samples by means of the physical vapour deposition (PVD) method at the Łukasiewicz-ITeE. Coating 2 (thickness: 4.2 µm) was made of TiN | TiCrN-AlCrN | AlTiCrN (counting from the substrate); Coating 3 (thickness: 4.4 µm) of TiN | TiCrN-AlCrN | AlCrTiN/Si_3_N_4_; and Coating 4 (thickness: 5.9 µm) of TiN | TiCrN-AlCrN | AlCrTiN/Si_3_N_4_ | AlCrTiSiN with an increasing oxygen gradient replacing nitrogen in the AlCrTiSiN outer layer. When selecting the groups of anti-wear coatings, the authors took into consideration the possibilities of their manufacturing at the Łukasiewicz-ITeE and the actual market demand for anti-wear coatings. The coating deposition process was carried out on a PLATIT Pi411 PLUS device (PLATIT AG, Selzach, Switzerland). The coating thickness was assessed with the Calotest method (Calowear CSM, Peseux, Switzerland). The coating structure is shown in Figure 1.

The coating design was predicated on the assumption that the TiN layer would enhance adhesion. The TiCrN-AlCrN intermediate layer served as the load-bearing component, exhibiting resistance to temperatures up to 900 °C. The external layers comprising AlTiCrN or AlCrTiN/Si_3_N_4_ were incorporated to evaluate the influence of the Si_3_N_4_ hard phase on high-temperature tribological performance. An additional external gradient layer, AlCrTiSiN+ON, was employed to assess the impact of oxygen within the outermost layer as a potential supplementary wear protection mechanism in elevated temperature environments. The main difference concerned the external layers. Four types of coating external layers were considered to interact with the externally introduced solid lubricant, i.e., a single structure: AlCrN, AlTiCrN, structure: AlCrTiN/Si_3_N_4_, and gradient structure: AlCrTiSiN+ON.

The layer composition of the samples was examined using a JY 10,000 RF Glow Discharge Optical Emission Spectrometer (GDOES) (Jobin-Yvon Horiba, Palaiseau, France).

Surface roughness was measured with the use of an optical profilometer (Talysurf CCI-Lite Non-contact 3D Profiler with TalyMap software (v.6.2.7487, 2015, https://www.taylor-hobson.com/ accessed on 10 October 2025, Taylor Hobson Ltd., Leicester, UK). Surface roughness Ra was accepted as an average of three measurements.

Coating adhesion tests employed a scratch method and were carried out on a REVETEST CSM Scratch Tester (Anton Paar GmbH, Graz, Austria) using a progressively increasing load in the range of 0–100 N, with a constant load growth rate of 10 N/mm. The Rockwell C type indenter with the angle of 120° and tip radius of 0.2 mm was used.

The hardness of the coatings was tested on an Anton Paar NanoHardness Tester (CSM) (Anton Paar GmbH, Graz, Austria). The measurements were carried out with the use of a Berkovich indenter (three-sided pyramid with a face angle of 65.27°) with the restricted mode of the maximum indenter penetration not exceeding 15% of the coating thickness. Nanoindentation hardness tests were performed by applying normal loads in the range of 5–10 mN. The Oliver & Pharr method was used to accurately calculate the hardness from the indentation load—displacement data.

### 2.3. Solid Lubricant Deposition

**Figure 2:**
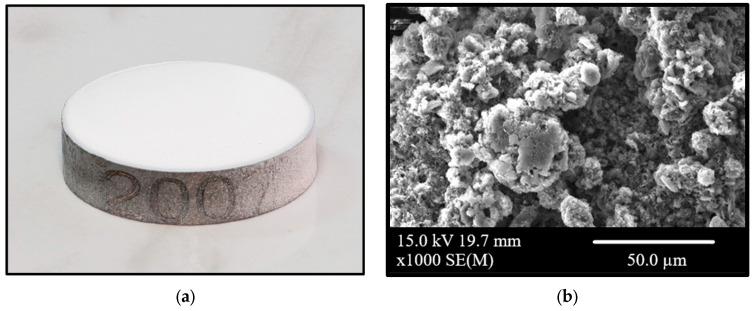
TiB_2_/Ti composite sample with hBN sprayed on the surface (**a**) and a magnification image of hBN particles obtained by means of scanning electron microscopy (**b**). Some grain agglomerates are visible.

An aerosol of the hexagonal boron nitride (HeBoCoat^®^ SL-E 200, Henze, Germany) was selected for an accessible deposition of a thin homogenous layer of a lubricant (Figure 2), which helps to obtain a reproducible layer on samples. Figure 2’s SEM image illustrates the surface characteristics of the uncoated composite with hBN sprayed on. The measured thickness of the boron nitride layer was c.a. 50 µm, determined using optical microscopy. To determine the time needed for the solvent to evaporate, FTIR spectra were recorded (FTIR-6200, Jasco, Hachioji, Japan, equipped with an Attenuated Total Reflectance (ATR) attachment).

**Figure 3:**
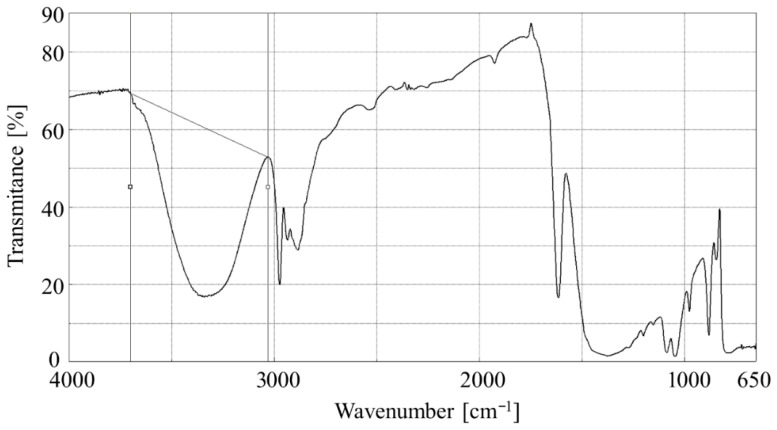
FTIR spectra obtained from the deposited hBN-aerosol with the marked integration range of the spectral band considered for the calculation of the evaporation kinetics.

The aerosol lubricant was sprayed on the ZnSe crystal. A series of analyses were conducted at 5 min intervals for up to 35 min. The observation of the solvent’s (i.e., ethyl alcohol) evaporation helped determine the kinetics by calculating the spectral band area at the wavenumber range of 3700–3030 cm^−1^ (stretching vibrations of the O-H bond in the alcohol hydroxyl group). The visual presentation of the integration range of the spectral band is shown in Figure 3.

**Figure 4:**
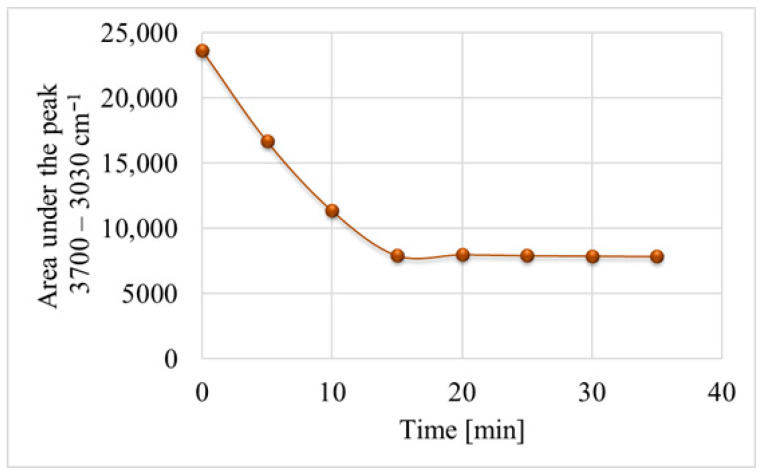
The kinetics of alcohol evaporation from the deposited hBN layer.

As shown in Figure 4, the solvent evaporation kinetics curve becomes a constant function after the 15th minute, which indicates a complete evaporation of the alcohol from the deposited layer.

### 2.4. Wear Test Methodology

**Figure 5:**
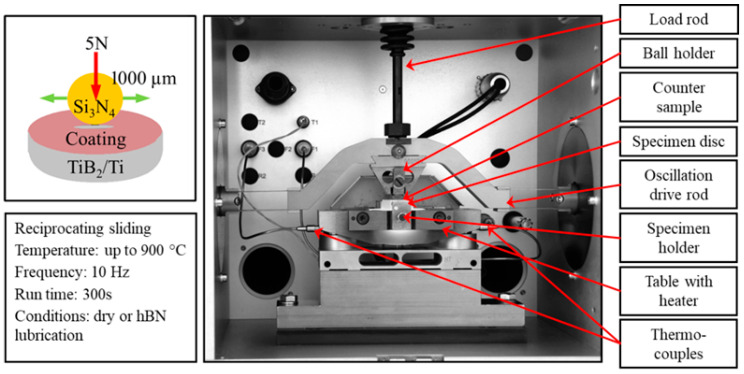
Ball-on-disc SRV oscillating tribosystem and tribocouple scheme.

The tribological tests were conducted on an SRV (Schwingung Reibung Verschleiβ [German for Reciprocating Friction Wear]) test machine (Optimol Instruments Prüftechnik GmbH, Munich, Germany)—a high-temperature reciprocating friction and wear tester (Figure 5). An electromagnetic drive was used to oscillate an upper counter-sample under normal load against a stationary test sample. A servomotor was used to apply the normal load. A fresh surface was used for each successive test.

In accordance with previous research [57], the following test parameters (Table 2) were applied. The test conditions were set between two boundaries: obtaining wear high enough to be clearly measurable while maintaining satisfactory repeatability, and avoiding the total removal of the investigated coating across the entire range of test temperatures, from room temperature (RT) up to 900 °C.

### 2.5. Surface Analysis Methods

Sample surfaces were examined before and after tribological tests by means of white light interferometry (WLI) (Talysurf CCI-Lite Non-contact 3D Profiler with TalyMap software (v.6.2.7487, 2015), Taylor Hobson Ltd., Leicester, UK). The obtained 3D surface topography images enabled the determination of the surface roughness and identification of the wear scar depth. The wear scars were characterised by images recorded with an SU-70 field-emission scanning electron microscope (FE-SEM, produced by Hitachi, Tokyo, Japan) and integrated with an energy dispersive spectrometer (EDS, produced by Thermo Scientific, Madison, WI, USA). The analyses were carried out under vacuum conditions of 1 × 10^−8^ Pa at an accelerating voltage of 15 kV, and a take-off angle of 30° with the use of a secondary electron detector (SE). The analysis of the elemental distribution responsible for the qualitative oxygen content on the observed surface was performed at 80× magnification for all wear scars obtained after the tribological tests.

The phase composition of the tested composite samples with an hBN lubricant sprayed on them was measured using a D8 Discover diffractometer (Bruker AXS GmbH, Karlsruhe, Germany), equipped with a Co X-ray tube (Kα1 = 1.79026 [M2] Å) to obtain information on lubricant phase transitions and its unit cell arrangement.

## 3. Results

### 3.1. Material Characterisation

**Figure 6:**
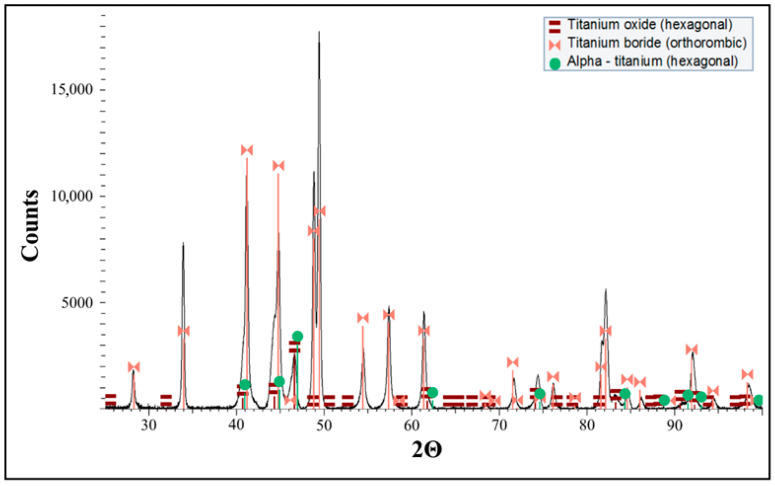
The XRD pattern of the TiB2/Ti composite material obtained by means of SPS.

The XRD pattern (Figure 6) contains titanium monoboride (orthorhombic), titanium (hexagonal), and titanium oxide (hexagonal). TiB presence is the result of a reaction between TiB_2_ and Ti that takes place during the SPS process. The absence of TiB_2_ indicates the reaction is completed.

Similar reactions were observed in the previous work [2], where the XRD patterns of the composite identified just a few peaks of TiB_2_, confirming the presence of an in situ formed TiB phase together with semi-reacted TiB_2_ particles in a titanium matrix containing both α (alpha) and β (beta) phases of titanium.

**Figure 7:**
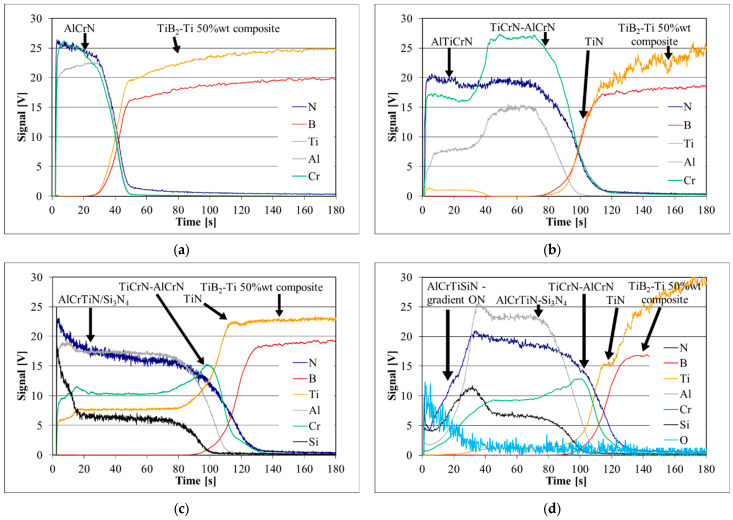
The glow-discharge optical emission spectroscopy (GDOES) depth profiles of the (**a**) Coating 1, (**b**) Coating 2, (**c**) Coating 3, and (**d**) Coating 4. The individual layers are indicated by arrows.

The depth profiles of the coatings deposited on the TiB_2_/Ti substrate obtained with GD-OES are presented in Figure 7. The GDOES profiles confirmed the presence of all layers depicted in Figure 1.

The depth profiles of the coatings confirmed the coating design presented in [61]. The TiN layer enhances adhesion. Then the TiCrN-AlCrN intermediate layer (Figure 7b–d) is deposited. The external layers are as follows: AlTiCrN (Figure 7b), AlCrTiN/Si_3_N_4_ (Figure 7c). A gradient layer AlCrTiSiN+ON is visible in Figure 7d.

**Figure 8:**
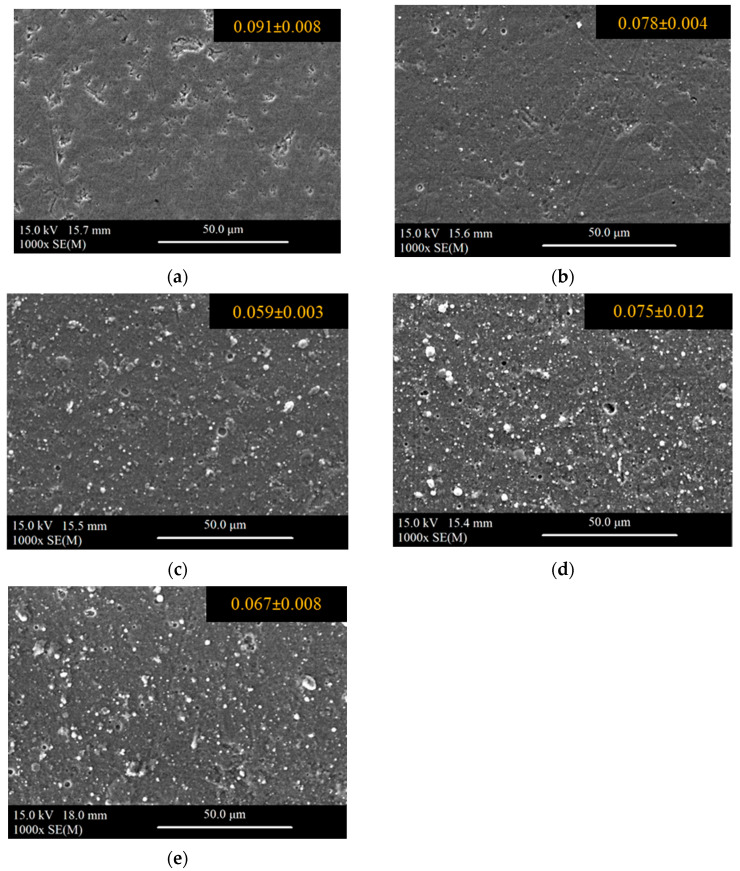
Samples surface SEM images with the Ra parameter (in µm): (**a**) uncoated composite, (**b**) Coating 1, (**c**) Coating 2, (**d**) Coating 3, and (**e**) Coating 4.

Surface roughness R_a_ was determined as an average of three measurements. Sample surface SEM images with the R_a_ parameter are shown in Figure 8. In each case, the composite surface R_a_ is below 0.1 µm. The deposition of coatings usually results in a slight reduction in surface roughness. Although the coatings cover the composite surface and the pores are closed, the surface is not free from numerous defects typical for PVD coatings.

**Figure 9:**
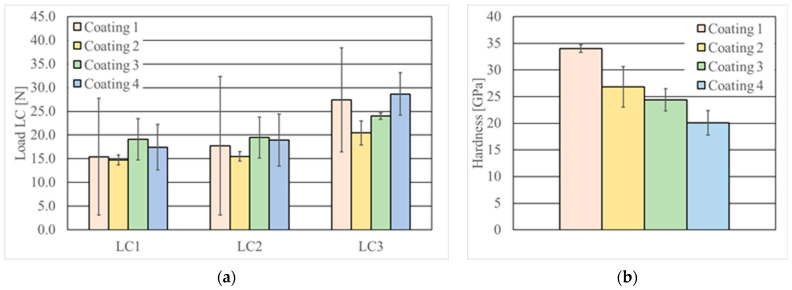
Coating properties: (**a**) adhesion (Rockwell C type tip), (**b**) hardness (Berkovich tip) results.

Adhesion results are summarised in Figure 9a. Three critical loads were determined for each coating (LC1, LC2, and LC3). The critical load LC1 denotes tensile brittle fracture and is considered to be the resistance to crack initiation. When loaded with LC2, the first signs of chipping and delamination appear on the scratch. The critical load LC3 is reached when the coating is completely removed and the substrate is exposed. The values of the forces causing the characteristic coating damage (cracking, chipping/spalling, breaking) were determined on the basis of the scratched area’s microscopic observations.

The previous studies [54] addressed the challenge of achieving strong adhesion between PVD coatings and TiB_2_/Ti composites. Due to the inherent porosity and the disparity in the mechanical properties of the constituent phases, obtaining a smooth surface presents significant difficulties. The scatter of the results is particularly pronounced in the single-layer Coating 1. In contrast, multilayer coatings incorporating a TiN interlayer (Coatings 2, 3, and 4) exhibited a significantly reduced scatter in the critical load results. Coatings 2 and 3 displayed lower LC3 than commercial Coating 1, but Coating 4 showed a higher adhesion than the commercial coating. Hardness (Figure 9b) does not correlate with LC3 value. The maximum hardness was revealed for Coating 1.

### 3.2. Wear Results

**Figure 10:**
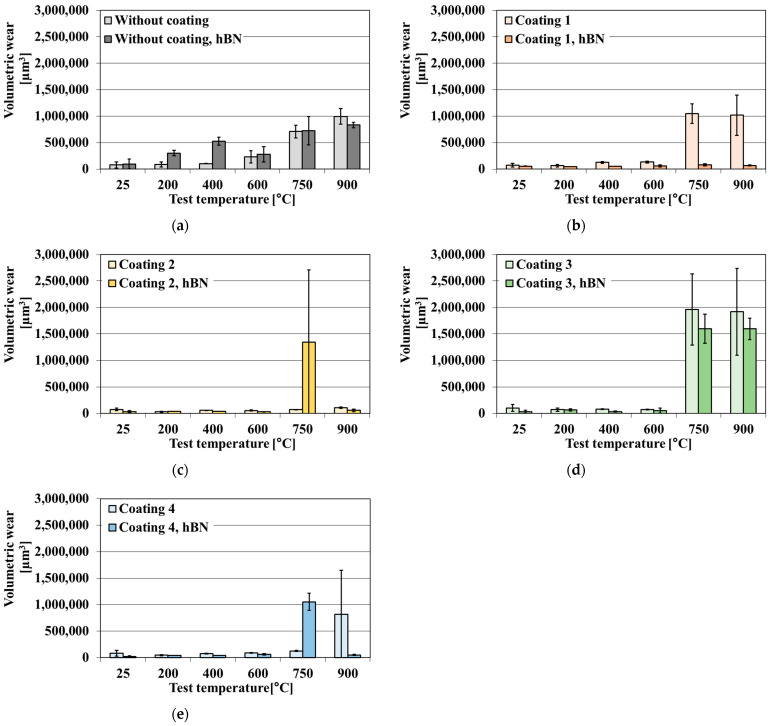
The volumetric wear of uncoated and coated samples of TiB_2_/Ti composite in the presence and absence of hBN lubricant. Tests conducted on SRV-type tester; the wear rate measured by means of optical profilometer. (**a**) Uncoated composite, (**b**) Coating 1, (**c**) Coating 2, (**d**) Coating 3, (**e**) Coating 4.

**Figure 11:**
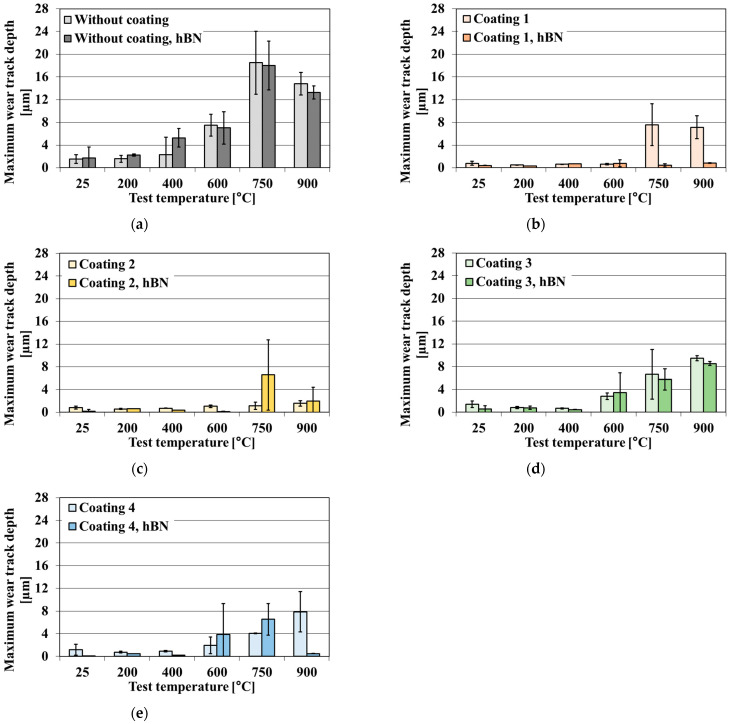
The maximum wear track depth of uncoated and coated samples of TiB_2_/Ti composite in the presence and absence of hBN lubricant. Tests conducted on SRV-type tester; the wear rate measured by means of optical profilometer. (**a**) Uncoated composite, (**b**) Coating 1, (**c**) Coating 2, (**d**) Coating 3, (**e**) Coating 4.

The volumetric wear and the maximum wear track depth of uncoated and coated samples of the TiB_2_/Ti composite in the presence and absence of the hBN lubricant are shown in Figure 10 and Figure 11.

The pure TiB_2_/Ti composite shows moderate wear at temperatures below 400 °C but reveals poor wear resistance under oscillating conditions (Figure 10a and Figure 11a) at temperatures exceeding 400 °C. The volumetric wear follows wear track depth. These results confirm the previous observations presented in [34], where the volumetric wear of the uncoated composite was stable up to 400 °C, but further temperature growth led to a doubling of volumetric wear at 600 °C and its 7-fold and 10-fold rises at 750 °C and 900 °C, respectively. The presence of a solid lubricant results in a higher volumetric wear and maximum track depth at test temperatures of up to 400 °C. The positive effect of the presence of hBN is visible at 900 °C, but, even then, the decrease in wear is not significant. Increasing the test temperature leads to a change from an abrasive to fatigue mode regime. Up to 20 µm deep holes typical of fatigue wear are formed in the wear scars at 750 °C and 900 °C. To sum up this tribo-couple behaviour, the application of an hBN lubricant to the Si_3_N_4_ ball and TiB_2_/Ti composite tribosystem does not improve wear resistance at a high temperature. These observations are consistent with [60], which demonstrated that TiB_2_/Ti composite materials exhibit limited compatibility with solid lubricants, even at room temperature.

Coating 1 on the TiB_2_/Ti composite improves its wear resistance from room temperature to 600 °C (Figure 10b and Figure 11b). When the hBN lubricant is added, the effect of wear reduction is more significant, especially at 750 and 900 °C—there is no rubbing through the coating. A synergetic wear resistance improvement is observed when double anti-wear protection is used at these temperatures. Coating 2 protects the composite from wear in the entire test temperature range—there is no rubbing through the coating (Figure 10c and Figure 11c). However, the addition of hBN impairs the behaviour of the Si_3_N_4_ ball and the coated TiB_2_/Ti composite tribocouple at 750 °C; the volumetric wear and maximum wear track depth increase several times. The deposition of Coating 3 improves wear resistance up to 600 °C (Figure 10d and Figure 11d). At 750 and 900 °C, the volumetric wear and maximum wear track depth increase significantly, and the coating is worn through. The application of hBN aggravates the wear factor at 600 °C. At 750 and 900 °C, hBN lubricant decreases (counter-sample—Coating 3 composite) tribo-couple volume wear, but not to the level of a pure composite. Coating 4 has the highest LC3 among the tested coatings. Its deposition raises wear resistance up to 750 °C (Figure 10e and Figure 11e). hBN application exacerbates the wear factor at 600 and 750 °C. However, at 900 °C it leads to reduced volumetric wear and maximum wear track depth. All the applied coatings increase the wear resistance of the TiB_2_/Ti composite, but only Coating 2 does so in the entire test temperature range. The application of the hBN lubricant leads to a synergistic effect with Coating 1 or an antagonistic effect at 600 and 750 °C with most of the remaining coatings. However, at 900 °C, in all cases, hBN reduces volumetric wear of the uncoated and coated composite.

**Figure 12:**
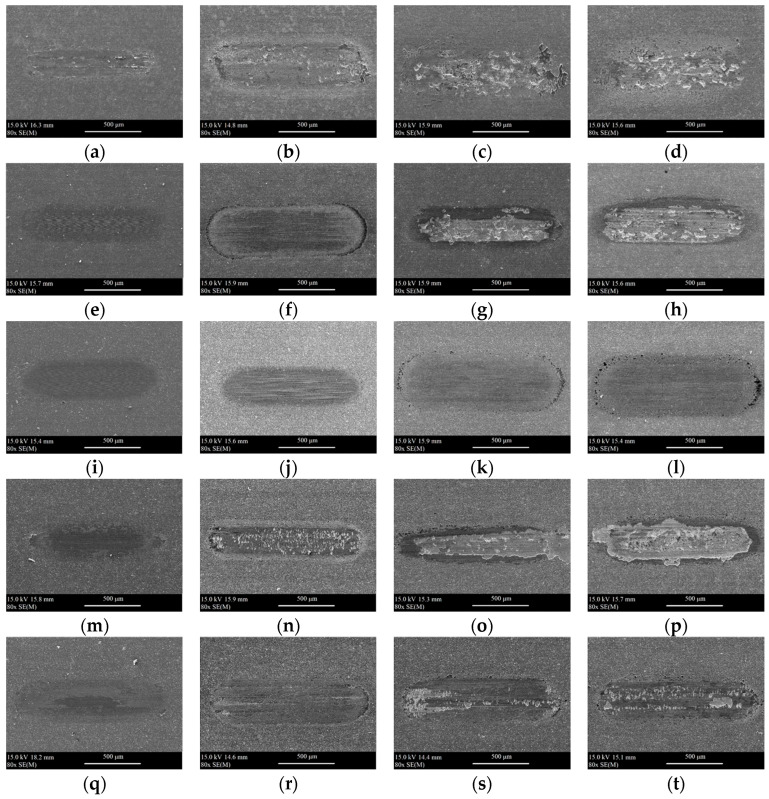
The SEM images of wear scars after tribological tests under dry conditions for the following samples: (**a**) uncoated 25 °C, (**b**) uncoated, 600 °C, (**c**) uncoated, 750 °C, (**d**) uncoated, 900 °C, (**e**) Coating 1, 25 °C, (**f**) Coating 1, 600 °C, (**g**) Coating 1, 750 °C, (**h**) Coating 1, 900 °C, (**i**) Coating 2, 25 °C, (**j**) Coating 2, 600 °C, (**k**) Coating 2, 750 °C, (**l**) Coating 2, 900 °C, (**m**) Coating 3, 25 °C, (**n**) Coating 3, 600 °C, (**o**) Coating 3, 750 °C, (**p**) Coating 3, 900 °C, (**q**) Coating 4, 25 °C, (**r**) Coating 4, 600 °C, (**s**) Coating 4, 750 °C, (**t**) Coating 4, 900 °C.

**Figure 13:**
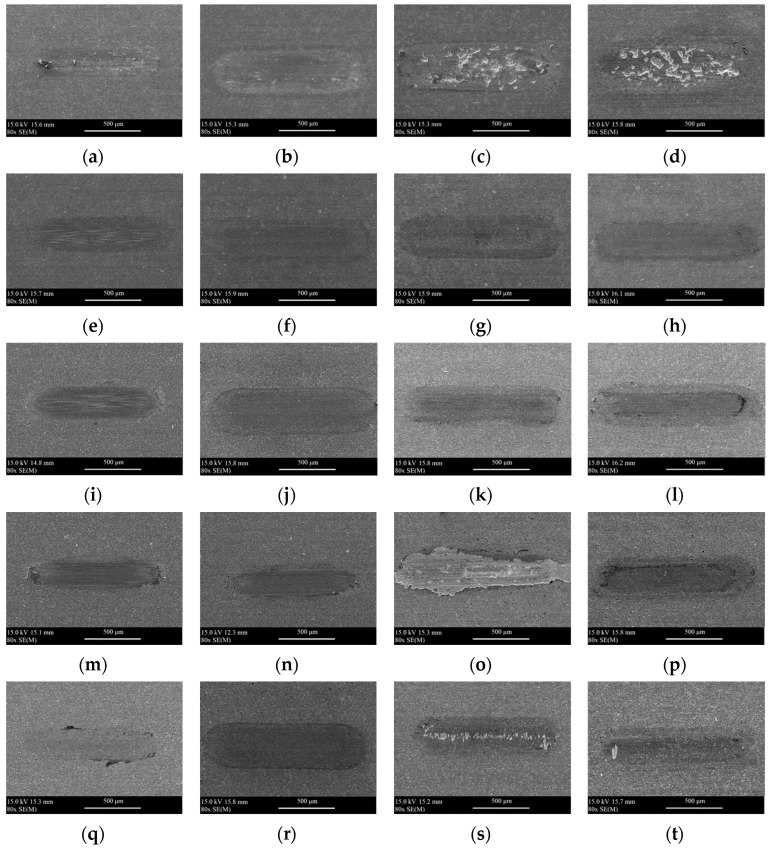
The SEM images of wear scars after tribological tests under lubricated conditions for the following samples: (**a**) uncoated, 25 °C, (**b**) uncoated, 600 °C, (**c**) uncoated, 750 °C, (**d**) uncoated, 900 °C, (**e**) Coating 1, 25 °C, (**f**) Coating 1 600 °C, (**g**) Coating 1, 750 °C, (**h**) Coating 1, 900 °C, (**i**) Coating 2, 25 °C, (**j**) Coating 2, 600 °C, (**k**) Coating 2, 750 °C, (**l**) Coating 2, 900 °C, (**m**) Coating 3, 25 °C, (**n**) Coating 3, 600 °C, (**o**) Coating 3, 750 °C, (**p**) Coating 3, 900 °C, (**q**) Coating 4, 25 °C, (**r**) Coating 4, 600 °C, (**s**) Coating 4, 750 °C, (**t**) Coating 4, 900 °C.

Figure 12 and Figure 13 show the SEM images of wear scars obtained after tribological tests under dry and lubricated conditions at 25 °C, 600 °C, 750 °C, and 900 °C.

The analysis of the SEM images shows the influence of temperature on the surface of the obtained wear scars. By covering the composite surface and closing the composite pores, the coatings protect the surface from deep cracking under oscillating motion and high pressure in the contact zone. A rapid progress of wear was observed for uncoated composite samples at 600–900 °C (for dry conditions) and 750–900 °C (for conditions with lubrication). A similar situation was observed for the samples of Coating 1, Coating 3, and Coating 4 at 750–900 °C under dry conditions. In the case of Coating 3 operating at 750 °C, the coating chipped. In the case of Coating 2, it neither chipped nor cracked. Only minor damage as a result of abrasive wear, both under dry and lubricated conditions, was observed.

**Figure 14:**
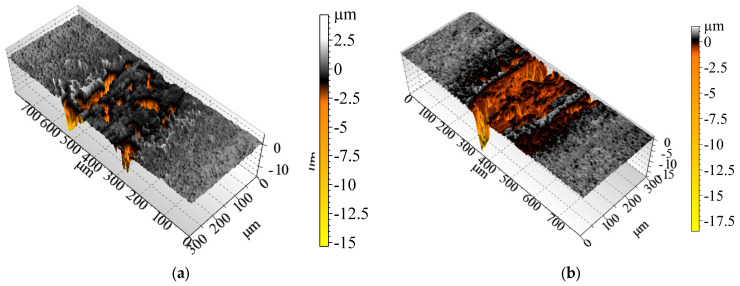

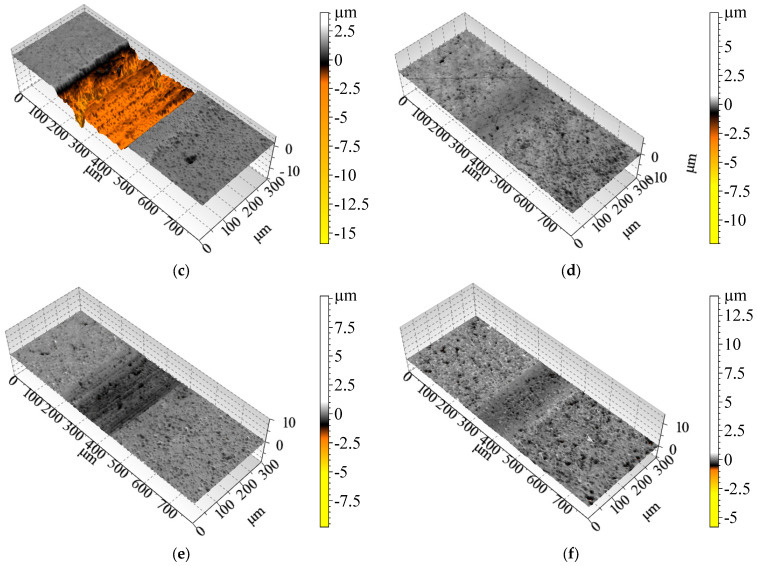
The 3D profiles of disc samples after tests at 900 °C: (**a**) uncoated composite, dry, (**b**) uncoated composite, hBN lubricated, (**c**) Coating 1, dry, (**d**) Coating 1, hBN lubricated, (**e**) Coating 2, dry, (**f**) Coating 2, hBN lubricated. Profiles made with TalyMap Platinium software, Taylor Hobson (v.6.2.7487, 2015).

Figure 14 shows 3D cross-section profiles of discs tested at 900 °C (a)—large-grained tear-outs can be seen on the uncoated samples. The addition of hBN does not lead to any improvement (b)—the wear scar is as deep as under dry conditions. Coating 1 does not protect the composite base at 900 °C (c); the coating is worn through, the wear scar is several µm deep. The coated tribo-pair working with an hBN lubricant (d) reveals a synergistic comparison—such a system protects the composite’s surface from severe wear at high temperatures. The deposition of Coating 2 protects the surface from severe wear at 900 °C (e). The application of hBN (f) has a low impact on wear scars, which have a similar depth as under dry conditions. At 900 °C, under dry and lubricated conditions, Coatings 3 and 4 have similar wear rates as Coating 1.

Table 3 presents the results of the EDS quantitative analysis of oxygen content on the surface of the analysed sample after tribological tests carried out at 25 °C, 600 °C, 750 °C, and 900 °C. In order to present the distribution of the analysed oxygen on the surface of the wear scars, distribution maps were made.

**Figure 15:**
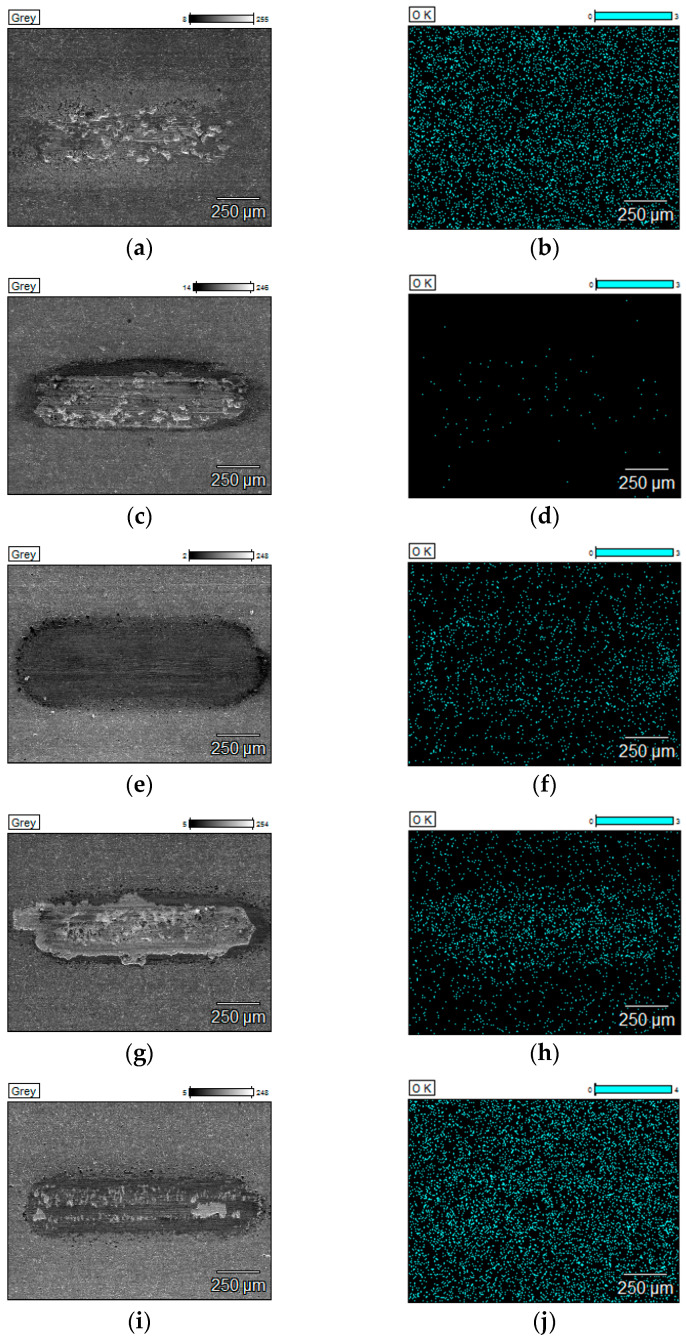
SEM images and EDS oxygen distribution maps after tests at 900 °C: (**a**) SEM image of uncoated composite, (**b**) EDS oxygen distribution map of uncoated composite, (**c**) SEM image of composite with Coating 1, (**d**) EDS oxygen distribution map of composite with Coating 1, (**e**) SEM image of composite with Coating 2, (**f**) EDS oxygen distribution map of composite with Coating 2, (**g**) SEM image of composite with Coating 3, (**h**) EDS oxygen distribution map of composite with Coating 3, (**i**) SEM image of composite with Coating 4, (**j**) EDS oxygen distribution map of composite with Coating 4.

**Figure 16:**
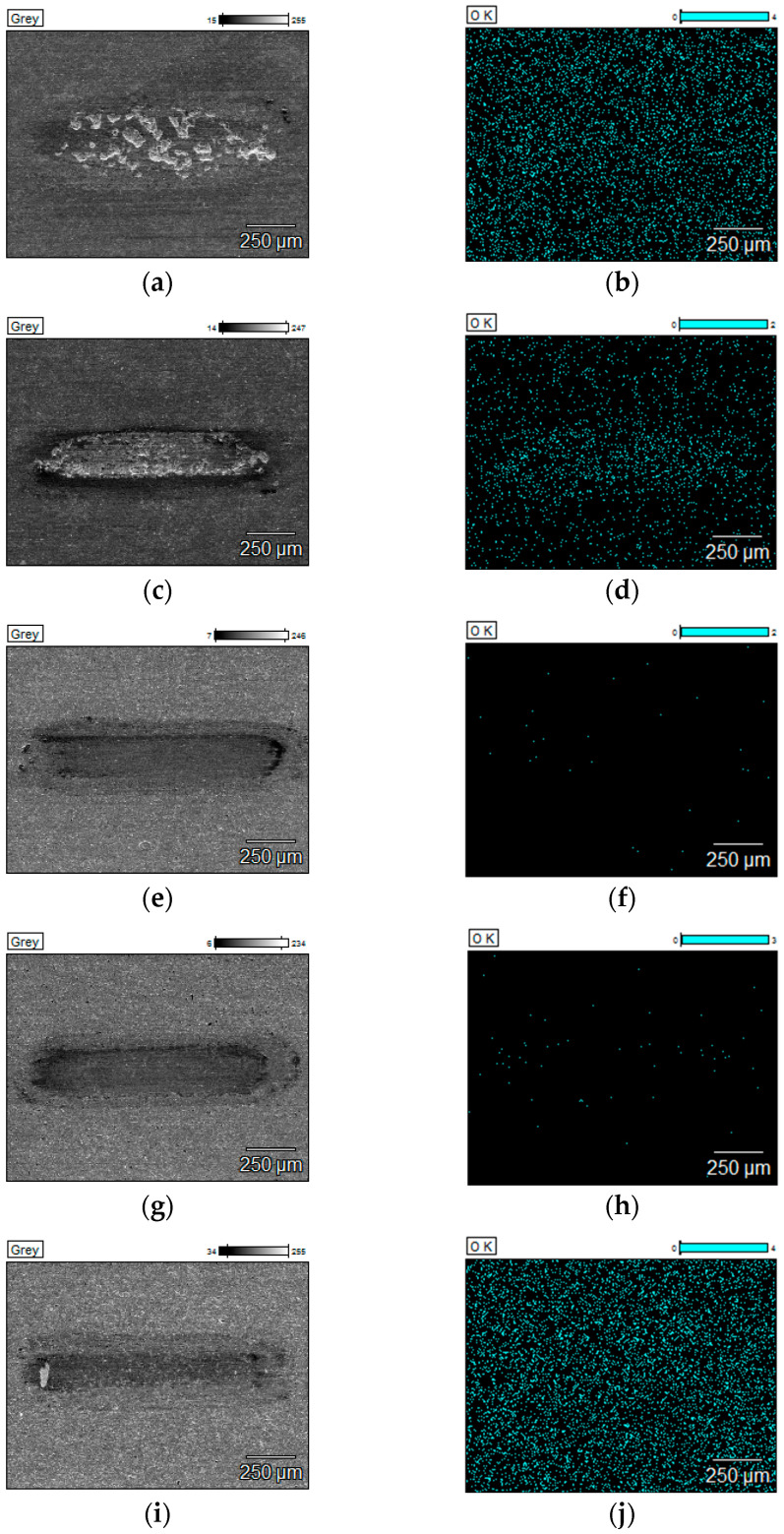
SEM images and EDS oxygen distribution maps after tests with hBN at 900 °C: (**a**) SEM image of uncoated composite, (**b**) EDS oxygen distribution map of uncoated composite, (**c**) SEM image of composite with Coating 1, (**d**) EDS oxygen distribution map of composite with Coating 1, (**e**) SEM image of composite with Coating 2, (**f**) EDS oxygen distribution map of composite with Coating 2, (**g**) SEM image of composite with Coating 3, (**h**) EDS oxygen distribution map of composite with Coating 3, (**i**) SEM image of composite with Coating 4, (**j**) EDS oxygen distribution map of composite with Coating 4.

Figure 15 and Figure 16 show the SEM images with a surface distribution of oxygen responsible for the qualitative composition of the sample surface after tribological tests carried out at 900 °C.

For the composite, the shift from mild to severe in wear regime as the temperature rises is highly likely intensified due to rapid surface oxidation. If the coating is rubbed through, the composite oxidation process is instant. In the case of the uncoated composite, temperature-dependent processes play a major role during wear: surface oxidation, grain tear-outs, and cracking propagation. In the case of Coating 4, oxygen is part of the outer layer of the coating, so the wear of the outer layer reduces the oxygen content in the wear track if the coating is not rubbed through. As a result, it is impossible to evaluate the oxidation level in the wear track in this case. All the deposited coatings protect the composite from oxidation or lower its surface oxidation level at high temperatures. However, in the case of Coating 2 and 3, oxidation rate is definitely lower than for other coatings. Coatings 2, 3, and 4 prevent the surface from deep cracking.

This study primarily focused on the distribution of oxygen within the wear tracks. Previous studies [35] indicated that Ti-TiB_x_ composite materials undergo oxidation, and TiO_2_ is detected in the wear scar at high temperatures. A more in-depth investigation into the formation and evolution of specific oxide phases during the friction of coated composites would constitute an interesting avenue for future research to elucidate the mechanism of high-temperature resistance enhancement by externally introduced hBN.

**Figure 17:**
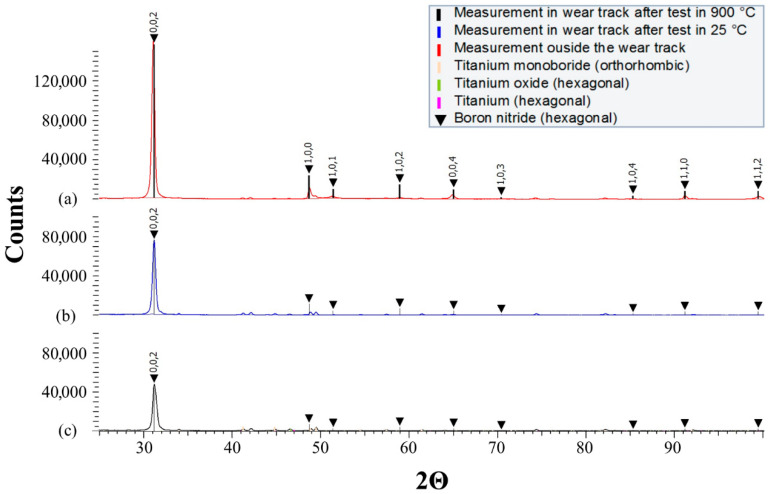
The XRD pattern of the TiB_2_/Ti composite material with hBN lubricant applied (**a**) red: measurement outside the wear track after the burnout of the lubricant carrier; (**b**) blue: measurement inside the wear track after testing at 25 °C; and (**c**) black: measurement inside the wear track after testing at 900 °C.

To analyse the behaviour of the hBN lubricant in the wear track phase, the composition of the tested composite samples with hBN sprayed on was measured by means of XRD. The measurements were carried out outside the wear track after the burnout of the lubricant carrier, then inside the wear track after testing at 25 °C, and inside the wear track after testing at 900 °C. All the obtained patterns (Figure 17) show peaks of low intensity, which indicates the composition of the substrate—titanium, titanium oxide, and titanium monoboride.

Inside and outside the wear tracks, differences in the intensity of the hBN peaks (black marks) can be observed, particularly for the hkl (002) peak, i.e., for the base plane of the unit cell. The intensity for (002) is significantly higher than the intensity of the other hBN peaks measured inside the wear scars, which almost disappear.

As illustrated in Figure 11, the use of hBN lubricant on coated surfaces significantly influences wear resistance, varying between different coatings. From the above-presented XRD patterns, it is evident that the presence of hBN plays a crucial role.

(002) is a privileged plane—the hBN phase is arranged so that the hexagonal planes of the unit cells are parallel to the sample surface. Easily sheared hexagonal layers are held together by weak van der Waals forces, leading to an easy breakage of chemical bonds and, consequently, low motion resistance. Such a plane arrangement, obtained during tribotests in wear scars, constitutes an optimal arrangement of the graphite-like lubricant to reduce friction and wear.

Based on the chemically inert nature of hBN, no reaction between the solid lubricant and coating materials is expected. The anti-wear action is possible through the presence of some hBN particles within the surface asperities in the friction zone. When the TiB_2_/Ti composite material is not protected by high-temperature wear-resistant coatings, the process of rapid removal of wear particles, along with hBN, occurs within the friction zone. In contrast, the surface of wear-resistant coatings, even at temperatures reaching 900 °C, creates potential reservoirs for hBN particles due to the presence of asperities and defects.

## 4. Discussion and Conclusions

The major challenge in the paper was ensuring satisfactory high-temperature tribological performance under oscillating motion and extreme contact pressure for TiB_2_/Ti composites manufactured by SPS. The achieved state-of-the-art understanding regarding the wear behaviour of various PVD coatings deposited on TiB_2_/Ti composites produced by SPS is presented. This investigation focused on lubrication with hexagonal boron nitride (hBN) as an external solid lubricant across a temperature range from ambient conditions up to 900 °C in frictional contacts subjected to extreme pressure and oscillating relative motion.

The results have proven that two factors should be taken into account to protect the TiB_2_/Ti composites against wear: the deposition of a coating and the presence of a solid lubricant.

In the current work, the investigated complex material consisted of the titanium composite with titanium boride reinforcement (TiB_2_/Ti composite) coated with PVD coating. The TiB_2_/Ti composite was protected by an AlCrN coating (1), TiN | TiCrN-AlCrN | AlTiCrN coating (2), TiN | TiCrN-AlCrN | AlCrTiN/Si3N4 coating (3), TiN | TiCrN-AlCrN | AlCrTiN/Si3N4 | AlCrTiSiN coating with an increasing oxygen gradient replacing nitrogen in the AlCrTiSiN outer layer (4), and an hBN solid state lubricant. The samples rubbing against the ceramic Si_3_N_4_ ball, were investigated in oscillating motion, at temperatures ranging from room temperature up to 900 °C, confirming that the coating and solid lubricant can present a synergetic or antagonistic effect on wear behaviour, depending on the coating material and temperature range.

The following detailed conclusions are drawn:

- TiB_2_/Ti composites are not recommended for use in friction pairs operating under oscillating motion at high temperatures exceeding 400 °C due to their susceptibility to significant wear. The obtained results indicate the following key factors influencing wear intensity: surface oxidation, crack propagation, and grain tear-out.
- The application of commercial AlCrN coatings to the surface of TiB_2_/Ti composites provides protection against surface oxidation, grain tear-out, and crack propagation at temperatures up to 600 °C.
- The best protection against wear and oxidation at higher temperatures (even up to 900 °C) was achieved for coatings when coating with AlCrN and AlTiCrN external layers and hBN lubricant were used simultaneously.
- It was observed that the coating and solid lubricant can have a synergetic or antagonistic effect on wear behaviour, depending on the coating material and the temperature range. The synergetic effect is observed for the hBN and AlCrN coating in the entire temperature range up to 900 °C, and for all coatings at 900 °C. The antagonistic effect was obtained for, e.g., Coating 2 (TiN | TiCrN-AlCrN | AlTiCrN) at 750 °C, Coating 3 (TiN | TiCrN-AlCrN | AlCrTiN/Si_3_N_4_) at 600 °C, and Coating 4 (TiN | TiCrN-AlCrN | AlCrTiN/Si_3_N_4_ | AlCrTiSiN with an increasing oxygen gradient in the outer layer coating).
- XRD results showed that inside the wear track after the test at 900 °C, the hBN phase is arranged in a way that the hexagonal planes of the unit cells are parallel to the sample surface. These planes arrangement is optimal to reduce friction and wear. Easily sheared hexagonal layers are held by weak van der Waals forces, which leads to easy breaking of chemical bonds and, as a consequence, to low motion resistance.

The objective of this paper was achieved. A high-temperature tribological performance was obtained in the range from room temperature to 900 °C in friction contacts under extreme pressure and with oscillating relative motion by application of PVD coatings deposited on TiB_2_/Ti composites manufactured by spark plasma sintering when lubricated with hexagonal boron nitride (hBN) as an external solid lubricant.

The Authors are aware of some limitations of their current research. Further research is required to establish the correlation between the coating characteristics (i.e., microstructures, compositions, and properties) and the tribological behaviour (i.e., wear depth, volume, and rate).

## Figures and Tables

**Table 1:** SPS process parameters applied to the TiB_2_/Ti composite sintering.

Sintering Temperature [°C]	Heating Rate [°C/min]	Uniaxial Pressure [MPa]	Sintering Time [s]
1250	100	50	900

**Table 2:** Selected SRV test parameters [57].

Parameter	Value
Test temperature [°C]	25, 200, 400, 600, 750, 900
Heating rate [°C/s]	1
Temperature stabilisation time [s]	1200
Load [N]	5
Stroke length [µm]	1000
Frequency [Hz]	10
Test duration [s]	300
Counter-sample ball diameter [mm]	10
Ball material [−]	Si_3_N_4_
Minimum number of repetitions	3

**Table 3:** The oxygen content on uncoated and coated disc samples at 25 °C, 600 °C, 750 °C, and 900 °C, measured by EDS.

Test Temperature [°C]	Out of the Wear Track		In the Wear Track		Out of the Wear Track		In the Wear Track	
	Average [at%]	Confidence [at%]	Average [at%]	Confidence [at%]	Average [at%]	Confidence [at%]	Average [at%]	Confidence [at%]
	TiB_2_/Ti composite				TiB_2_/Ti composite, hBN			
25	0.0	0.1	47.4	15.9	0.0	0.1	50.9	5.6
600	44.9	3.0	59.7	1.6	45.5	1.8	39.3	7.2
750	58.5	0.9	57.2	5.7	57.1	2.2	56.8	3.5
900	63.8	0.5	65.3	1.4	61.0	1.6	63.4	2.3
	Coating 1				Coating 1, hBN			
25	0.6	1.3	0.4	0.9	0.4	0.9	0.0	0.1
600	0.3	0.7	4.7	2.7	1.9	2.0	4.3	4.5
750	2.8	1.5	53.7	3.5	0.5	0.6	13.9	3.3
900	1.1	1.4	60.1	1.8	1.7	0.8	7.5	2.9
	Coating 2				Coating 2, hBN			
25	0.6	0.4	0.5	1.0	1.4	1.7	1.5	1.5
600	4.1	1.1	0.5	0.7	4.7	2.4	0.7	0.9
750	5.6	2.4	7.2	0.7	5.8	2.6	6.2	2.0
900	4.9	2.7	15.5	2.5	3.7	1.8	13.6	7.1
	Coating 3				Coating 3, hBN			
25	0.2	0.5	0.9	1.6	3.8	1.4	9.8	17.1
600	3.8	1.5	45.2	2.3	11.8	1.2	5.1	3.0
750	10.8	1.6	58.9	3.5	10.0	3.1	59.7	0.6
900	10.9	6.0	61.4	3.8	12.4	2.7	15.4	4.0
	Coating 4				Coating 4, hBN			
25	44.3	0.5	34.6	16.8	45.8	1.0	44.9	0.7
600	43.4	2.0	37.6	3.2	44.6	1.0	17.6	4.8
750	45.5	0.9	46.4	3.7	45.8	0.5	50.9	0.4
900	45.8	0.5	50.4	6.6	44.6	0.7	47.0	1.2

## Data Availability

The original contributions presented in this study are included in the article. Further inquiries can be directed to the corresponding author.
